# Death at sea—the true rate of occupational fatality within the Australian commercial fishing industry

**DOI:** 10.3389/fpubh.2022.1013391

**Published:** 2022-10-14

**Authors:** Greg Penney, William Byrne, Marcus Cattani

**Affiliations:** School of Medical and Health Sciences, Edith Cowan University, Perth, WA, Australia

**Keywords:** Fishing, Safety, occupational, coroner, Australian, fatality

## Abstract

Although the safety performance of the Australian commercial fishing industry has been the subject of multiple investigations, it has ultimately remained undefined. While most Australian industries notify industry regulators of significant workplace incidents and injuries in their operations, the majority of persons in the commercial fishing industry are contractors who are paid piecework and in some jurisdictions specifically excluded from the worker compensation legislation, meaning that most occupational injuries, including fatalities, are not captured in the centralized worker compensation data sets. This study presents the analysis of a systematic review of industry databases, published academic, and, Australian coroners reports to assist improve the definition of the nation's commercial fishing industry safety performance. The analysis shows occupational fatality rates are significantly higher than currently reported, and recurring factors contributing to deaths at sea remain unaddressed. The study is significant as it demonstrates how workplace injuries and deaths can be hidden within data sets applying broad industry classification and provides a foundation for future research in Australian fishing and other industries.

## Introduction

The Australian Commercial Fishing Industry (ACFI) directly employs approximately 6,000 persons. On average, the industry produces 174,000 tons of product per year, with an economic value of $5.3 bn, representing a strong contribution to the Australian economy (1). The industry is diverse with a wide range of vessel types, fishing techniques, and is geographically spread around much of the 36,000 km of Australian coastline (2). Employment related to wild catch fishing is estimated at between 5,600 and 7,500 people in recent seasons (3, 4), with an average of 4,292 full-time and 1,675 part-time employees per year over the period.

Although the detail of Australian occupational health and safety legislation varies between its States and Territories, henceforth referred to as States, the general principles are based on the United Kingdom's Robens-style legislation (5). The occupational health and safety legislation requires organizations to implement a risk-based approach to eliminate or minimize risks as so far as is reasonably practicable and create a safe workplace, which is regulated by a State government agency. In addition, each State has workers' compensation legislation which provides a no-blame insurance-based system to provide income and rehabilitation assistance, including medical and other expenses associated with workplace injuries (6). Each State reports worker compensation summary data and information, including fatalities, to Safe Work Australia which compiles and reports regularly. The annual report includes comparison and trends over time between States, industries, hazards, and interventions (7). Safe Work Australia works with States and industry to set the agenda for performance improvement and inform national policy (8).

However, the commercial fishing industry regulation is not consistent and comprises a complex mix of Federal and State agencies, with legislation specific to geographic regions, the species being fished, licensing and operation of vessels, and the prevention of illegal fishing in Australian waters. While fishing vessels are considered a workplace consistently across all jurisdictions legislation, the people who work on them may not be deemed to be workers or employees. The classification of workers is dependent upon the applicable occupational health and safety, and worker's compensation legislation (9–18).

For example, The Western Australian Workers' Compensation and Injury Management Act 1981 (10) defines a worker as follows:

“*any person who has entered into or works under a contract of service or apprenticeship with an employer, whether by way of manual labor, clerical work, or otherwise and whether the contract is expressed or implied, is oral or in writing”*.

However, this Western Australian Act specifically excludes crews of fishing vessels as these workers:

“*in respect to injuries occurring to such members of a fishing vessel as contribute to the cost of working that vessel, and are remunerated by shares in the profits or the gross earnings of the working of that vessel*”.

Similarly elsewhere, such as in Queensland, which has the largest fishing fleet, crews of fishing vessels do not receive salary or wages, instead their income is a share of the profits, or loss, of the vessel, after running costs and other liabilities are deducted. In effect, this “share catch” income arrangement results in the workers being self-employed contractors in their workplace, and they personally are responsible for paying their tax, superannuation (i.e., pension), and frequently medical and other insurances (19, 20).

In effect, the exclusion of fishing crew being classified as a worker removes most obligations on the employer to report workplace incidents and injuries to the workers/crew. Crew may of course report workplace injuries to their insurer; however, this may have an impact on the cost of the insurances. These circumstances have created systemic under-reporting of incidents and injuries in the commercial fishing industry.

Safe Work Australia categorizes the worker compensation data and information using the Australian and New Zealand Industrial Classification (ANZSIC). As ANZSIC coding groups “business units carrying out similar productive activities” together (21), commercial fishing, farming, and agriculture are collectively considered one group and are reported as such in occupational databases and reports (7, 8, 22–24).

Safe Work Australia (7) reports agriculture, forestry, and fishing in the top two industry classifications from 2003 to 2018 for total fatalities per year, with transport, postal, and warehousing having the highest total number of fatalities for 7 of the 16 years analyzed.

In 2004, agriculture, forestry, and fishing recorded 77 fatalities, the highest number observed from the data reviewed. In 2019, the lowest number, 30 fatalities, was recorded. Agriculture, forestry, and fishing recorded the highest fatality rate per 100,000 workers for 15 of the 16 years, peaking at 21.6 fatalities per 100,000 workers in 2004 and recording the lowest in 2019 of 9.1 fatalities per 100,000 workers. As Safe Work Australia reports the fishing industry data combined with agriculture and forestry, analysis of fishing-specific data is not possible.

Safe Work Australia reports rates of injury and fatality per 100,000 workers regardless of injury or size of industry. Noting that the ACFI only has a workforce of approximately 6,000 people (1), we hypothesized that reporting of injuries and fatalities in this manner dilutes and hides the true contextualized rates of fatality in the ACFI. Furthermore, we posit that the rates of occupational fatality in the industry may be significantly higher than currently acknowledged in existing reporting. The combination of employment arrangements, lack of inclusive legislative requirements to report occupational injuries, and the collective grouping of commercial fishing with other industries in occupational statistical databases give rise to the problem statement: “*the true state of safety performance within the Australian commercial wild catch fishing industry remains unknown*”.

International literature and data sets (25–27) report commercial fishing as one of the world's most dangerous occupations with reoccurring factors of causation. It is therefore hypothesized that the contextualized rate of occupational fatality within the ACFI is higher than currently reported, and reoccurring causes of injury and death are present. To answer this question, this research aims to answer the following:

1. What is the contextualized rate of occupational fatality within the Australian wild catch commercial fishing industry?
2. What are the recurring contributing factors of occupational fatality within the Australian wild catch commercial fishing industry?

This study aims to answer these questions and improve understanding of the true state of safety within the ACFI. We attempt to do this through two separate systematic literature reviews of (1) published research focussed on the ACFI and (2) coronial investigations and other safety investigations and reports relevant to the ACFI with results presented according to the Preferred Reporting Items for Systematic Reviews and Meta-Analyses (PRISMA) checklist. The results are then distilled to identify the number of fatalities within the industry with greater accuracy, while thematic analysis is completed to identify the recurring contributing factors.

The motivation for, and significance of, the study is 2-fold. First, the results of the study may assist the development of targeted safety interventions within the ACFI and the reduction of avoidable fatalities within the industry. Second, the findings with regards to the suitability of current data classification and analysis may have broader-reaching impacts across Australian workplaces wherever broad industry data classifications have the potential to disguise or dilute actual injury and fatality rates.

The study is subsequently presented as follows. First, we present the methodology of the study and detail the search terms, inclusion criteria, and limitations of the systematic literature reviews. Next, we present the results of each review before discussing their implications and recommending potential industry improvements. Finally, we provide our conclusion and recommendations for future research.

## Methodology

In the first phase of the study, a systematic review of contemporary studies on occupational health and safety within the ACFI was completed. This systematic review enabled the identification, examination, and synthesis of relevant academia, government, and industry reports. The second phase of the study involved a systematic review and thematic analysis of Australian coronial findings and safety investigations related to ACFI incidents. This narrative approach was selected due to both the anticipated heterogeneous and limited research available, as well as enabling a review of the ‘state of knowledge’ of the field (28).

For Phase 1, studies meeting the following criteria were included in the analysis: peer-reviewed studies or reports, as well as post-incident reviews, inquiries, and inquests after incidents, published by government and non-government organizations (in Australia, industry research is completed by both sectors). Two review authors independently tested the search criteria and completed the initial search before reviewing the titles and abstracts, and selecting final articles for detailed full-text analysis. Any disagreement was resolved by discussion and majority decision between all article authors. Following the removal of duplications, the titles and abstracts were screened. The results are presented according to the Preferred Reporting Items for Systematic Reviews and Meta-Analyses (PRISMA) checklist.

The search strategy included only terms relating to the occupational health and safety of crew on fishing vessels in the Australian wild catch fishing industry (Table 1). A secondary search of bibliographies identified further literature for inclusion. Completed in September 2020, the review included English-language papers published in the last 20 years (2001–2020) to ensure currency of evidence. Seminal papers from outside the date range were considered for inclusion where appropriate. Databases included Medline, ProQuest, Scopus, Web of Science, and Google Scholar. Non-English-speaking literature, abstracts, citations, thesis, unverified or unsubstantiated press or news media reports, and articles that are not related to occupational safety of crew on fishing vessels in the Australian wild catch fishing industry were excluded. A review of the “gray literature” in Google was subsequently completed using the same search terms (Table 1). This literature review was informed by a consideration of industry literature, policy and non-peer-review professional journals or publications, and non-medical media.

**Table 1:** Search terms.

Sources	Medline, ProQuest, Scopus, Web of Science, and Google Scholar
Search terms	Australia^*^AND fish^*^AND (industry OR commercial) AND (fatal^*^ OR death OR safe^*^ OR health)
Limits	English Language AND Published Between 2001–2020

The symbol * indicate standard nomenclature in searches.

For Phase 2, as the databases reviewed were specific to Australian incidents within the relevant jurisdictions, all available reports related to commercial fishing were screened. Databases included Australian Coroners Courts in each State (i.e., Australian Capital Territory “ACT”, New South Wales “NSW”, Northern Territory “NT”, Queensland “QLD”, South Australia “SA”, Tasmania “TAS”, Victoria “VIC”, and Western Australia “WA”) including all coronial published inquiries, findings, and reports into deaths, as well as safety investigations and reports by the Australian Transport Safety Bureau (ATSB) and Australian Maritime Safety Administration (AMSA). The search was completed in September 2020. Two review authors independently tested the search criteria and completed the initial search before reviewing the titles and abstracts and then selecting final articles for detailed full-text analysis. Any disagreement was resolved by discussion and majority decision between all authors. Following the removal of duplications, the titles and abstracts were screened. The results are presented according to the Preferred Reporting Items for Systematic Reviews and Meta-Analyses (PRISMA) checklist.

### Calculating contextualized fatality rates

Contextualized fatality rates (*F*_*c*_), that is the fatality rate within each industry per 10,000 Australian workers as opposed to fatality rate per 100,000 Australian workers, will be calculated using equation (1):


(1) $$\begin{array}{l} F_{c}=F_{t}/W_{t} \end{array}$$

where *F*_*t*_ is the number of fatalities reported over the time period assessed in years; and *W*_*t*_ is the average number of workers in the industry during the same time period.

## Results

**Figure 1:**
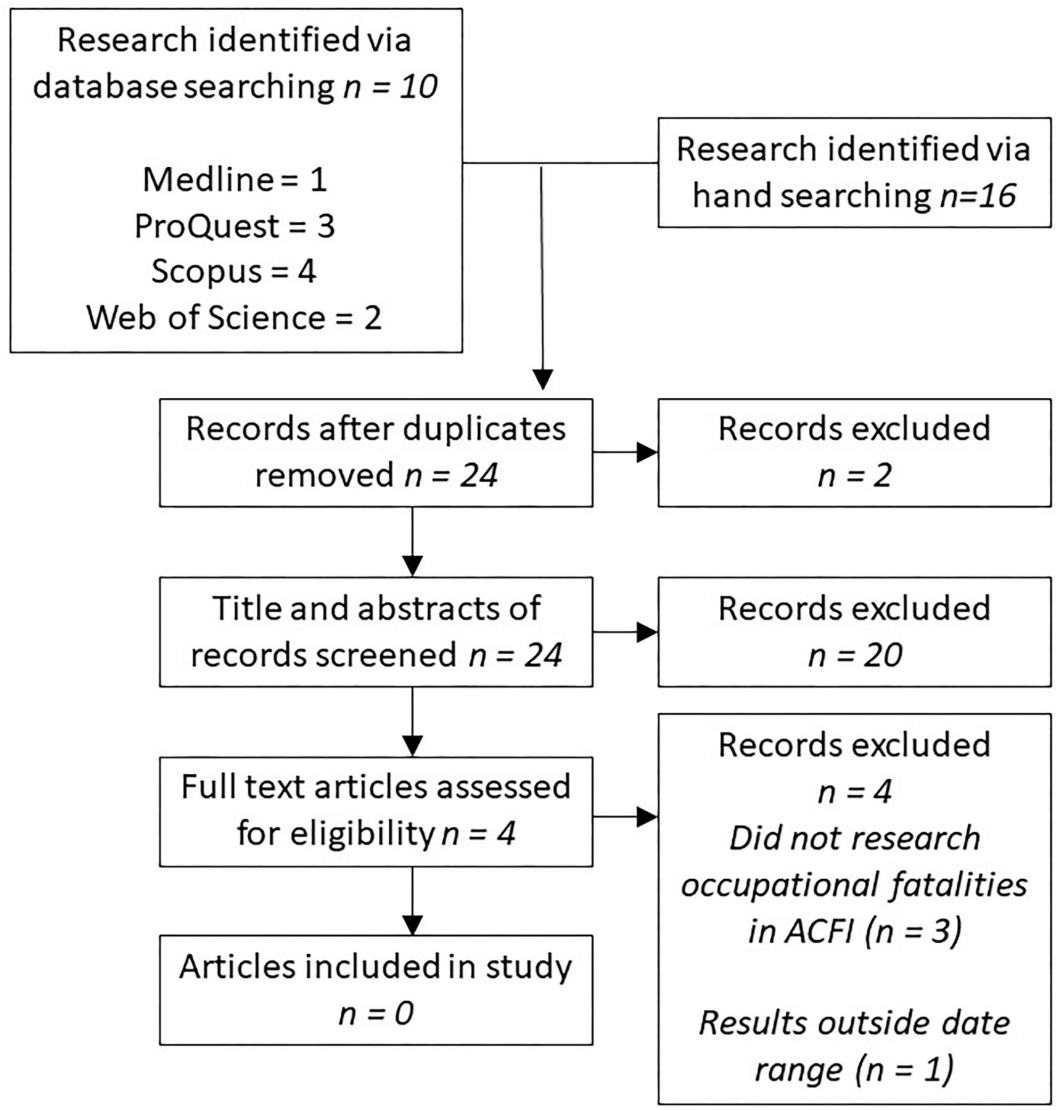
Phase 1 systematic literature review of peer-reviewed academia presented in accordance with PRISMA guidelines.

The search in Phase 1 (Figure 1) yielded only four studies suitable for full review in the study. Of these works, only one explored work-related fatalities and was deemed potentially suitable for inclusion in the review; however, the date range examined in the study was 1989 to 1992 and was subsequently excluded. No academia was therefore identified as being suitable for inclusion in the study. Handsearching “gray literature”, that is industry reports, initially identified 13 possible results, ultimately yielding five reports suitable for inclusion in the study (8, 29–32).

Industry literature (29, 31) reports higher ACFI fatality rates than those reported by Safe Work Australia (23). Extracted from Safe Work Australia data of occupational fatalities, Brooks (29) reported 14 occupational fatalities between 2003 and 2010. Confirming the limitations of using occupational health and safety reporting to accurately calculate incidents within the ACFI previously discussed in this paper, Lower (31) subsequently extracted data directly from the National Coroners Information System (NCIS) and reported 55 occupational fatalities between 2003 and 2013 (33 occupational fatalities between 2003 and 2010 in comparison with Brooks' figures). By comparison, DMIRS (30) reports four fatalities between 2009 and 2019 in the Fishing, Hunting, and Trapping industry subdivision, but does not specify which of these incidents (if any) are attributable to wild catch fishing. Both the remaining texts, the Commonwealth of Australia, report “*They never came home*” (8) and Lyons “*Best Practice Review of Workplace Health and Safety Queensland*” (32) cite ANZSIC division data that does not differentiate between agriculture, forestry, and fishing.

Chronological analysis of the data reported by Lower (31) and categorization by both mechanism of fatality, and wild catch fishing industry sector, is detailed in Table 2. Collectively, drowning accounts for 75% of all fatalities, with capsize (including capsizing as a probable result of nets being caught) being a substantial contributor to fatalities, accounting for 29% of all deaths. Prawn fishing is the most dangerous known sector, accounting for 25% of fatalities during the period. On average, there are 5.5 fatalities annually across the sector for the period. Unfortunately, neither exposure nor task level analysis and description were provided across the different industry sectors within the literature reviewed or within the ANZSIC database from which the industry sectors are drawn. This subsequently prevents more detailed analysis or description within this study.

**Table 2:** ACFI fatalities by mechanism of injury 2003–2013 [Data from (31)].

**Mechanism of death**	**Finfish trawling**	**Line fishing**	**Marine**	**Marine n.e.c**	**Prawn fishing**	**Rock lobster**	**Total**
Boat fire—drowning					1		1
Diving—shark attack				2			2
Drowning—not otherwise classified			4		1		5
Drowning—capsize	4			3	2		9
Drowning—fall overboard	2	2	4	2	2	1	13
Drowning—entangled in net			3				3
Drowning—nets caught, capsizing boat			2		5		7
Drowning—scuba related			1				1
Drowning—washed overboard				1			1
Drowning—diving & entangled				1			1
Drowning—collision at sea					1		1
Electrocution		1	1				2
Entanglement	2						2
Fire				2			2
Head injury—waves						2	2
Pully / winch					1		1
Tractor					1		1
Unknown				1			1
Total	8	3	15	12	14	3	55

^*^Note—the “Marine” classification is the overarching category and is used when specific detail of the sector is unknown from the data. The “Marine (nec)” classification is used when another type of known fishing has been involved but is not listed.

**Figure 2:**
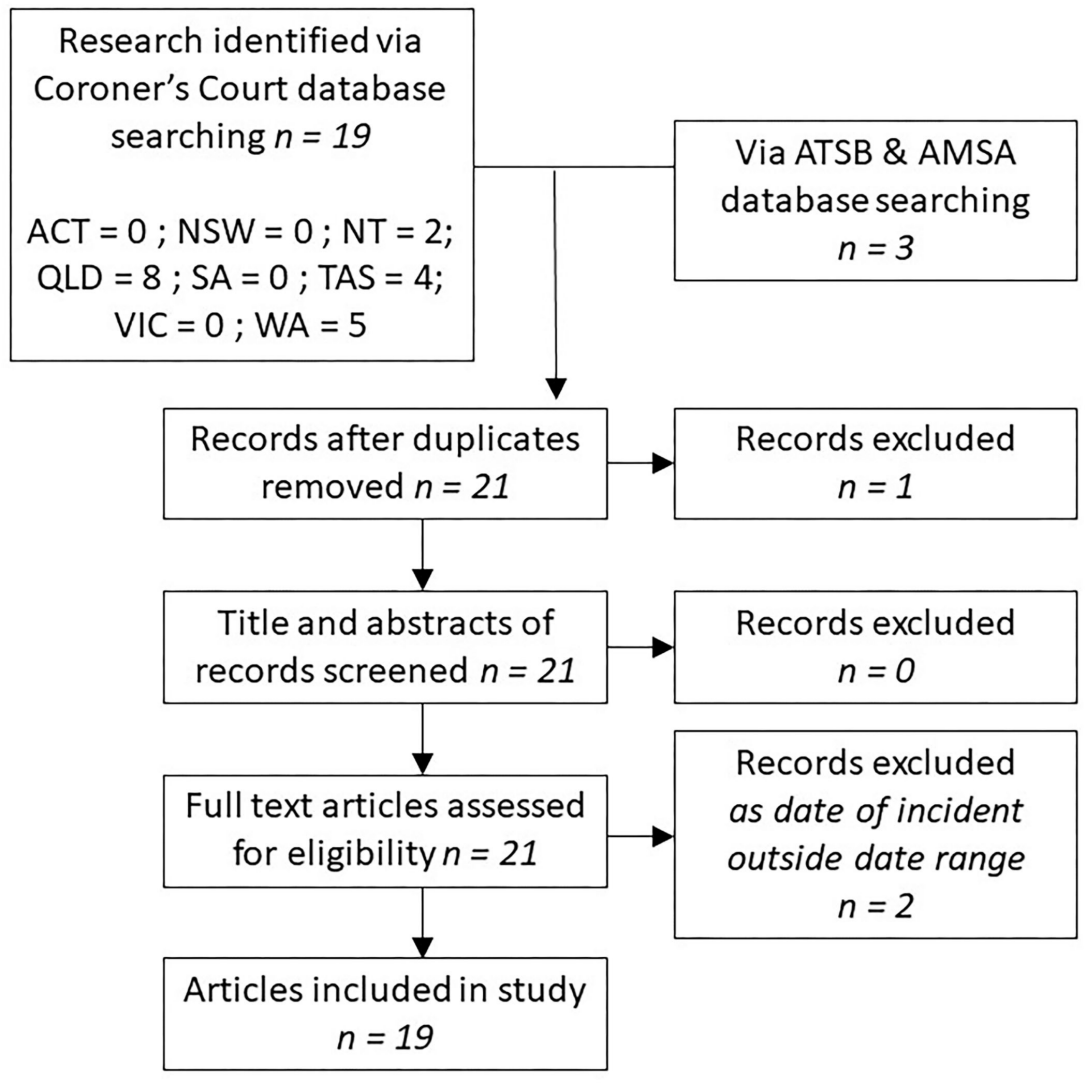
Phase 2 systematic literature review presented in accordance with PRISMA guidelines.

The search in Phase 2 identified 22 reports for inclusion, 19 from coronial inquiries and three ATSB reports (Figure 2). One duplication was identified (i.e., one coronial and ATSB report investigated the same incident), and two reports related to incidents occurring prior to 2001. These three reports were excluded, resulting in 19 reports included in the study. Analysis of findings that matched the search criteria is summarized in Table 3. The issues of vessel stability, lack of action by regulators, and lack of enforcement of safety regulations were recurrent, particularly in cases of multiple fatalities.

**Table 3:** Coronial finding and ATSB report summary.

**Year**	**Mechanism of death**	**Notes**	**Reference**
2018	Sea snake bite	Neurotoxic venom, prawn trawler, remote location	D0164/2018
2016 & 2017	Capsize and drowning	Vessel stability, significant modification detrimental to stability post required testing. Multiple fatalities	COR 2016/1622, 2016/1637, 2017/4709, 2017/4711, 2018/5398, 2018/5402, 2018/5405, 2018/5407.
2016	Fell overboard and drowned	Work accident	1572/2016
2015	Capsize and drowning	Vessel stability, non-compliance with regulations, failure of regulators, substantial modifications to the extent “it would have been considered a new vessel” para 282. Multiple fatalities	1190/2015; 1191/2015; 11,036/2015
2013	Capsize and drowning	Vessel sea worthy—unknown cause of capsize.	2013/2509
2013	Traumatic head injury	Work accident	Cooper (2017)
2013	Electrocution	Noncompliance with Work Health and Safety legislation, previous coronial recommendations for similar death, confusing regulatory regime, lack of response by regulators	D210/2013
2012	Drowning	Drowning secondary to air embolism during dive operations	6,008-2012
2009	Capsize and drowning	Nets hooked. 13 year old vessel compliant with stability requirements at time of constructed, not tested since. Multiple fatalities.	16/08/2012; 04/09/2012; 05/09/2012
2006	Capsize and drowning	Poor safety attitude, lack of union safety protection, lack of response by regulators	COR 2012/05(6)
2006	Fell overboard and drowned	Recommendation regulators to make EPIRB and Personal Floatation Devices mandatory	892/06(8)
2004	Capsize and drowning	Vessel stability, nets hooked, lack of response by regulators	COR-632/05(8)
2003	Drowning post collision with bulk carrier	Drowning post collision with bulk carrier	ATSB 195
2001	Drowning	Drowning post arm being caught in rope and being dragged overboard.	4,066/01

Fatality rates within the ACFI per 10,000 workers as a comparison against the other highest-ranking Australian industries are calculated using equation 1 and are detailed in Table 4. These figures indicate that average occupational fatality rates per 10,000 workers in Australian commercial fishing, calculated at 9.2, are higher than Agriculture and Road Freight Transport by a factor of 2.8 and 3.8, respectively, and higher than Construction by a factor of 30.7. The calculated rate of 9.2 fatalities per 10,000 within the ACFI is significantly higher than the peak rate of 21.6 fatalities per 100,000 workers in 2004 (equivalent to 2.2 fatalities per 10,000 workers) reported by Safe Work Australia (23).

**Table 4:** Contextualized fatality rates per 10,000 workers.

**Industry**	**Workforce**	**Fatalities**	**Fatalities per 10,000** **workers in that** **industry**
Fishing	6,000^a^	5.5^b^	9.2
Agriculture	231,415^c^	76.6^b^	3.3
Road freight transport	142,808^d^	34^e^	2.4
Construction	1050,000^f^	35^g^	0.3

a average workforce within limits reported (3, 33).

b average fatalities over the period reported (31).

c average workforce from 2011 to 2016 calculated from (34).

d (35).

e (22).

f (36).

g average annual fatalities 2003–2018 (23).

## Discussion

The absence of peer-reviewed academic research into occupational injuries and fatalities within the ACFI was unexpected by the research team and is itself concerning, especially given the reputation of the industry internationally as being one of the most dangerous in the world (26, 27). In his synthesis of 16 international fishing industry case studies, Knapp (26) concluded that fishing was the world's most dangerous occupation, and both effective regulation and safety improvements could only be achieved when the extent of the problem is understood. Within the ACFI, this is not the case, and the true rate of occupational injury remains unknown. Estimating the expected number of workplace injuries in the ACFI, and how this compares to the data held by existing databases of workplace injuries held by the Australian Government remains problematic. The omission of ACFI occupational injuries from worker compensation-based data sets, combined with the lack of regulatory enforcement within the industry, results in little, if any, available data (37). This issue is not unique to Australia, with Maritime New Zealand (38) and McGuiness et al. (27) acknowledging that significant under-reporting within the industry is common across the globe. Accordingly, this suggests that the ACFI has remained relatively under-scrutinized from an academic perspective and is largely informed by industry reports and government data sets which do not appear to provide an accurate representation of the state of the industry.

The first research question can therefore only be partially answered with any certainty. Based on the available data, the average occupational fatality rate per 10,000 workers in Australian commercial fishing is calculated at 9.2, almost 4.2 times higher than the peak rate of 2.2 reported by Safe Work Australia (23). This demonstrates the inclusion of commercial fishing in the same ANZSIC Division A coding as agriculture and forestry is misleading as it significantly dilutes the actual fatality rates within the industry. Significant discrepancy is evident between stated national occupational health and safety data sets and actual fatality rates within the ACFI. In turn, this has the potential to misdirect national safety priorities and regulatory reforms that data should be used to guide decision makers. We noted these data sets were referenced in “*They never came home—the framework surrounding the prevention, investigation and prosecution of industrial deaths in Australia*” (8) which makes recommendations regarding the strategic direction of national occupational health and safety initiatives across Australian industry.

The second research question can also be answered with limited certainty. The issues of vessel stability, lack of action by regulators, and lack of enforcement of safety regulations were recurrent, particularly in cases of multiple fatality. As with other issues within the ACFI, they appear consistent with commercial fishing internationally (26, 27). Comments of Magistrate O'Connell [39, para 2] summarize the sentiment across the multiple inquiries within the ACFI (39):

“*the circumstances are a significant concern as 18 commercial fishermen have died at sea in the waters off Queensland in the years since 2004. Too many persons in the fishing and trawling industry have been lost over the years and despite a number of inquests recommending improved safety measures little has actually changed or been implemented despite technology being available*”.

The conclusion of Judge Cavanagh [40, para 1] was particularly damning, stating (40)

“*In my view, the evidence at this inquest has highlighted the unacceptable and indeed the shameful state of workplace safety on large numbers of Australian domestic fishing vessels. The lack of regulation and enforcement by authorities is of great concern*”.

The reasons for these factors remaining unaddressed within the industry may not only be as a result of incomplete and invalid data, but also due to the unique employment arrangements within the ACFI that fail to promote worker protection. As Barnes 2006, [41, p9] articulates (41),

“*in other dangerous industries, unions have successfully lobbied for legislation to reduce the risks to workers so that when anybody enters a mine or a building site they are required to wear steel capped boots and hard hats. In the fishing industry where many of the workers have limited education and other employment opportunities and unionism is almost non-existent, a level of risk that would not be tolerated in shore based jobs is the norm*”.

The primary limitation of this study is also one of the strengths and key findings. The lack of reliable and valid data with which to make robust conclusions impeded the ability of the study to accurately compare ACFI injury rates with other occupational groups. At the same time, this finding is significant as it demonstrates that existing Australian occupational health and safety data sets do not recognize impacts of employment arrangements within the ACFI on the validity of occupational injury and fatality statistics. Future research investigating ACFI injury rates through industry-specific structure surveys and injury analysis similar to studies conducted in Norway (27) may in part provide an indication of these rates.

Three limitations of all national safety reporting in Australia identified in the course of this study are the following:

1. potentially misleading unit of fatality or injury per 100,000 workers across the Australian workforce;
2. use of ANZSIC Division coding for data analysis by Safe Work Australia which broadly classifies industry groups as opposed to ANZSIC Group coding; and
3. the lack of alignment between report narratives and the coded pattern of injury in the reports analyzed.

To improve the safety performance of the ACFI informed by a comprehensive incident and injury data set, we recommend the following:

1. develop an estimate of the contextualized rates of occupational fatality and injury, informed by an analysis of the industry incident and injury reports and reporting, and structured with the ANZSIC coding system;
2. Recognize contractors who are paid piecework as workers, and giving them the same protection in the workplace as other Australian workers, across all legal jurisdictions;
3. Engage with industry stakeholders including employers and Regulators concerning the prevention of high consequence incidents such as those involving vessel stability (e.g., regulator workplace inspections and stability checks);
4. Assess the pros and cons of reporting occupational fatality and injury rates per 10,000 workers in an industry, as opposed to diluting the rates using the 100,000 workers; and
5. Informed by the above, review the Safe Work Australia priority industries league table, and associated performance improvement initiatives.

## Conclusion

The aims of this research were to determine the contextualized rate of fatality and to identify recurring contributing factors of occupational fatality within the Australian wild catch commercial fishing industry. Through the application of a systematic literature review of peer-reviewed academia, industry reports, coronial documents, and the critical review of industry-specific data, these aims have been achieved, albeit with limited certainty.

Using industry contextualized fatality rates, commercial fishing in Australia (excluding aquaculture) is the most dangerous Australian occupation with a contextualized average annual fatality rate of 9.2 fatalities per 10,000 workers. By comparison, the next two highest industries identified were Agriculture (3.3 fatalities per 10,000 workers) and Road Freight Transport (2.4 fatalities per 10,000 workers).

However, the true rate of contextualized injury cannot be determined due to a lack of valid and robust industry data reported *via* the States' Workers Compensation regulator to Safe Work Australia. It appears this situation is caused by the specific exclusion in the legislation of fishing crew as workers, thereby removing the fishing crew employer's obligation to report incidents and injuries.

Multiple and extensive coronial investigations have not only repeatedly acknowledged the fishing industry as a highly dangerous occupation, but also have found vessel instability, lack of action by regulators, and lack of enforcement of safety regulations were recurrent themes, particularly in cases of multiple fatality.

Further research is required to determine the true state of safety within the Australian wild catch commercial fishing industry. We recommended this research should focus on attitudes toward reporting within the ACFI; adoption of coronial recommendations; impacts of safety interventions; and vessel safety and regulatory compliance.

## Data availability statement

The original contributions presented in the study are included in the article/supplementary material, further inquiries can be directed to the corresponding author.

## Author contributions

GP was the lead researcher and primary contributer. WB and MC both provided a substantial contribution to the paper. All authors contributed to the article and approved the submitted version.

## Funding

Fisheries Research and Development Corporation 2017-231: to develop a national marine safety extension resource toolkit and to trial with all fisheries jurisdictions is supported by funding from the FRDC on behalf of the Australian Government.

## Conflict of interest

The authors declare that the research was conducted in the absence of any commercial or financial relationships that could be construed as a potential conflict of interest.

## Publisher's note

All claims expressed in this article are solely those of the authors and do not necessarily represent those of their affiliated organizations, or those of the publisher, the editors and the reviewers. Any product that may be evaluated in this article, or claim that may be made by its manufacturer, is not guaranteed or endorsed by the publisher.

## References

[1] BDO EconSearch. Australian Fisheries Aquaculture Industry 2017/18: Economic Contributions Data Summary Framework. Adelaide, SA: BDO EconSearch (2020). Available online at: https://www.frdc.com.au/sites/default/files/products/2017-210%20Data%20Summary%20and%20Framework%20Report.pdf

[2] Government of Australia. Border Lengths – States and Territories. Available online at: https://www.ga.gov.au/scientific-topics/national-location-information/dimensions/border-lengths (accessed September 5, 2020).

[3] Department of Agriculture. Department of Agriculture Employment. (2017). Available online at https://www.agriculture.gov.au/abares/research-topics/fisheries/fisheries-and-aquaculture-statistics/employment-2017 (accessed September 5, 2020).

[4] Mobsby D Koduah A. Australian Fisheries and Aquaculture Statistics 2016. Canberra: Australian Bureau of Agricultural and Resource Economarine incidents and Sciences. (2017).

[5] Johnstone R Tooma M. Work Health and Safety Regulation in Australia : the model act. Federation Press. (2012).

[6] Dunn CE Thakorlal S. CCH Australia Limited. Australian master work health and safety guide (2nd ed.). Australia: CCH Australia Limited. (2014).

[7] SafeWork Australia. Work-related Traumatic Injury Fatalities, Australia. Canberra: Safe Work Australia. (2020).

[8] Australia, Commonwealth of They never came home—the framework surrounding the prevention investigation investigation and prosecution of industrial deaths in Australia. (2018).

[9] *Occupational Health and Safety (Maritime Industry) Act, (Government of Australia 1993)*. Commonwealth Government of Australia (1993). Available online at: https://www.ilo.org/dyn/natlex/docs/ELECTRONIC/40673/101306/F1206142060/AUS40673.pdf

[10] *Worker's Compensation and Injury Management Act 1981*. Government of Western Australia (2018). Available online at: https://www.legislation.wa.gov.au/legislation/prod/filestore.nsf/FileURL/mrdoc_40999.pdf/$FILE/Workers%20Compensation%20And%20Injury%20Management%20Act%201981%20-%20%5B12-b0-00%5D.pdf?OpenElement

[11] Workers Rehabilitation and Compensation Act 1988. Tasmania: Government of Tasmania (2015).

[12] Workplace Injury Rehabilitation and Compensation Act 2013. Victoria, BC: Government of Victoria (2013). Available online at: https://content.legislation.vic.gov.au/sites/default/files/2022-09/13-67aa046%20authorised.pdf

[13] Workplace Injury Management Workers Compensation Act 1998. Sydney, NSW: Government of New South Wales (2022). Available online at: https://legislation.nsw.gov.au/view/html/inforce/current/act-1998-086

[14] *Workers' Compensation and Rehabilitation Act 2003*. Government of Queensland (2022). Available online at: https://www.legislation.qld.gov.au/view/pdf/inforce/current/act-2003-027

[15] *Work Health and Safety Act 2012*. Adelaide, SA: Government of South Australia (2015). Available online at: https://www.publicsector.sa.gov.au/__data/assets/pdf_file/0009/218853/002-Work-Health-and-Safety-Act-2012.pdf

[16] *Work Health and Safety (National Uniform Legislation) Act 2011*. Government of the Northern Territory of Australia (2020). Available online at: https://legislation.nt.gov.au/Legislation/WORK-HEALTH-AND-SAFETY-NATIONAL-UNIFORM-LEGISLATION-ACT-2011

[17] *Work Health and Safety Act 2011*. Government of Australian Capital Territory (2018). Available online at: https://www.legislation.gov.au/Details/C2018C00293

[18] *Occupational Health and Safety (Maritime Industry) Act, (Government of Australia 1993)*. Commonwealth Government of Australia (1993). Available online at: https://www.ilo.org/dyn/natlex/docs/ELECTRONIC/40673/101306/F1206142060/AUS40673.pdf

[19] WorkCoverQLD. Covering the crew of a fishing vessel for workers' compensation. (2018). Available online at: https://www.worksafe.qld.gov.au/agriculture/articles/covering-the-crew-of-a-fishing-vessel-for-workers-compensation. (accessed November 21, 2020).

[20] WorkCoverQLD. Who should I cover? (2019). Available online at: https://www.worksafe.qld.gov.au/insurance/which-insurance-product-is-right-for-you/accident-insurance/who-should-i-cover (accessed November 21, 2020).

[21] ABS (1993) 1292.0 - Australian and New Zealand Standard Industrial Classification (ANZSIC), 1993. Australian Bureau of Statistics. Available online at: https://www.abs.gov.au/ausstats/abs@.nsf/66f306f503e529a5ca25697e0017661f/5BD72C7D74F64C6BCA25697E0018FD27?opendocument (accessed November 22, 2020).

[22] SafeWork Australia. Fatalities and serious injuries in the Road Transport Industry. Canberra: Safe Work Australia. (2017). Available online at: https://www.safeworkaustralia.gov.au/system/files/documents/1707/road-transport-infographic.pdf (accessed September 7, 2020).

[23] SafeWork Australia. Work-related Traumatic Injury Fatalities, Australia. Canberra: Safe Work Australia. (2018).

[24] *WorkSafe occupational injury dataset*. WorkSafe Australia (2019).

[25] Chauvin C Bouar GL Lardjane S. Analysis of occupational injuries in the sea fishing industry according to the type of fishery and the fishing activity. Int Maritime Health. (2017) 68:7. 10.5603/IMH.2017.0006 28357834

[26] Knapp G. International commercial fishing management regime safety study: synthesis of case reports. (2016). Available online at: http://www.fao.org/3/a-i5552e.pdf (accessed September 5, 2020).

[27] McGuinness E Aasjord H Utne I Holmen IM. Injuries in the commercial fishing fleet of Norway 2000–2011. Safety Sci. (2013) 57:18. 10.1016/j.ssci.2013.01.008

[28] Baumeister R Leary M. Writing narrative literature reviews. Rev Gen Psychol. (1997) 1:311–20. 10.1037/1089-2680.1.3.311

[29] Brooks K. Health and safety in the Australian fishing industry. Prahran East VIC: Rural Industries Research and Development Corporation. (2011).

[30] Department of Mines, Industry Regulation and Safety. (2020). State of the Work Environment, Work-related traumatic injury fatalities in Western Australia 2009-2010 to 2018-2019. DMIRS, Perth. Available online at: https://www.commerce.wa.gov.au/sites/default/files/atoms/files/sowe_fatalities_2018-2019.pdf (accessed September 10, 2020).

[31] Lower T. Mapping Work Health and Safety risks in the Primary Industries. Moree NSW: Rural Industries Research and Development Corporation. (2015).

[32] Lyons T. Best Practice Review of Workplace Health and Safety Queensland - Final Report. (2017). Available online at: https://www.worksafe.qld.gov.au/__data/assets/pdf_file/0016/143521/best-practice-review-of-whsq-final-report.pdf (accessed September 10, 2020).

[33] Mobsby D Koduah A. Australian Fisheries and Aquaculture Statistics 2016. Canberra: Australian Bureau of Agricultural and Resource Economarine incidents and Sciences. (2017).

[34] Department of Agriculture. Profiles of Australian fisheries - Department of Agriculture. (2020) Available online at: https://www.agriculture.gov.au/abares/research-topics/fisheries/fisheries-and-aquaculture-statistics/profiles-of-australian-fisheries#northern–territory (accessed September 5, 2020).

[35] IBIS. Road Freight Transport in Australia - Market Research Report. BISWorld (2020).

[36] AI Group. Australia's Construction Industry: Profile Outlook. July 2015. AI Group. (2015) Available online at: http://cdn.aigroup.com.au/Economarineincidentc_Indicators/Construction_Survey/2015/Construction_industry_profile_and_Outlook.pdf (accessed September 7, 2020).

[37] Cavanagh G. Inquest into the death of Ryan Harry Donoghue. (2013).

[38] Maritime New Zealand. Annual Report 2016/17. Auckland: Maritime New Zealand. (2017).

[39] Department of Agriculture. Key Trends, Global Context and Seafood Consumption – 2017 Report. Commonwealth Government of Australia (2017).

[40] Barnes M. Inquest into the Suspected Death of Rodney John Baker COR-632/05(8). Queensland Coroners Court (2006).

[41] O'Connell D. Joint Inquest into the presumed deaths of David Barry Chivers and Matthew Neil Roberts from the FV Cassandra and Adam Jeffrey Bidner and Zachary John Feeney and Christopher David Sammut and Eli Davey Tonks from the FV Dianne and the deaths of Adam Ross Hoffman and Benjamin Patrick Leahy from FV Dianne. Queensland Coroners Court (2019).

